# Characterization of the complete mitochondrial genome of the tea leaf roller *Caloptilia theivora* (Insecta: Lepidoptera: Gracillariidae)

**DOI:** 10.1080/23802359.2019.1624647

**Published:** 2019-07-11

**Authors:** Shi-Chun Chen, Hong-Yan Jiang, Jing Shang, Xiang Hu, Ping Peng, Xiao-Qing Wang

**Affiliations:** Tea Research Institute of Chongqing Academy of Agricultural Science, Chongqing, P. R. China

**Keywords:** Mitochondrial genome, *Caloptilia theivora*, Gracillariidae, tea pest

## Abstract

The tea leaf roller, *Caloptilia theivora* (Walsingham), is a serious pest of tea plants. We have obtained and annotated the complete mitochondrial genome of *C. theivora* (GenBank accession No. MK541932). The entire mt genome is 15,297 bp long with an A + T content of 80.66%. The mt genome of *C. theivora* encodes all 37 genes that are typically found in animal mt genomes, consists of 13 protein-coding genes, 2 ribosomal RNA, and 22 transfer RNA genes. The gene order is consistent with other moths mt genome in Ditrysia. The control region of this genome is 192 bp long with a high A + T content of 96.35%, and located between the *rrnS* and *trnI* genes. Phylogenetic analysis was performed using 13 protein-coding genes among 19 moths showed that *C. theivora* is closely related to species of Gracillariidae.

The tea leaf roller, *Caloptilia theivora* (Walsingham), belonging to the family Gracillariidae in the order Lepidoptera, is a serious pest of tea plants in China and Japan (Kamimuro et al. 2017, 2018). The larvae feed on the leaves of *Camellia sinensis, C. japonica*, *C. sasanqua*, and *C. theifera*. They sneak the tender leaf or roll the young leaves into triquetrous insect buds for hiding themselves and feeding. The fecal particles accumulated in the insect buds, causing pollution to the leaves and influenceing the tea quality. In this study, we obtained and described the complete mitochondrial genome of *Caloptilia theivora* for the first time. The larvae of *C. theivora* were collected from a tea plantation at Yongchuan, Chongqing, China, in 2016, and identified to species by morphology and sequence of *cox1* (Nakadai and Kawakita 2016). Voucher specimens (#CQNKY-LE-04-01-01) were deposited at the Insect Collection, Tea Research Institute of Chongqing Academy of Agricultural Science, Chongqing, China.

The complete mt genome of *C. theivora* is a typical closed-circular and double-stranded DNA molecule in size of 15,297 bp (GenBank accession MK541932). The overall nucleotide composition of the major strand of the tea leaf roller mt genome is as follows: A = 40.99% (6,271), C = 11.43% (1,748), G = 7.92% (1,211) and T = 39.66% (6,067), with a total A + T content of 80.66%, that is heavily biased toward A and T nucleotides. AT– and GC-skew of the whole J-strand of *C. theivora* is 0.017 and –0.181, respectively. The mt genome of the tea leaf roller encodes all 37 genes usually found in animal mt genomes, including 13 protein-coding genes, 2 ribosomal RNAs, and 22 transfer RNAs. The control region is 192 bp long and located between the *rrnS* and *trnM* genes. The A + T content of this region is 96.35%, the highest level of each region in this mt genome. The gene arrangement of this mt genome is conserved as other moths mt genome in Ditrysia. Twenty-three of all 37 genes are encoded on the majority strand (J-strand) and the others encoded by the minority strand (N-strand). Twelve of the 13 PCGs start with ATN codons (ATG for *atp6*, *cox2*, *nad1*, *cob*, *nad4*, and *nad4L*; ATT for *atp8*, *nad2*, *nad5*, and *nad6*; ATA for *cox3* and *nad3*) and *cox1* used CGA as start codon, same situation exists in most Lepidoptera species (Chen et al. 2016). Three PCGs (*cox1*, *cox2*, and *nad4*) have incomplete terminal codons consisting of single T nucleotide, and the other PCGs stop with TAA and TAG. The nucleotide length of tRNA genes is ranging from 61 bp (*trnA*) to 80 bp (*trnS_1_*), and A + T content is ranging from 70.42% (*trnK*) to 89.39% (*trnD*). All of the 22 tRNA genes have the conventional cloverleaf-shaped secondary structure. The two rRNA genes have been identified on the N-strand in the *C. theivora* mt genome: the *rrnL* gene locates between *trnL_1_* and *trnV* genes, and the *rrnS* gene between the *trnV* gene and the control region. The length of *rrnL* and *rrnS* genes was 1389 and 770 bp, and their A + T content was 85.46% and 85.84%, respectively.

We analyzed amino acid sequence of 13 PCGs with the maximum likelihood (ML) method to understand the phylogenetic relationship of *C. theivora* with other moths. The mt genome sequence of *Drosophila melanogaster* (GenBank accession no. DMU37541) was used as an outgroup. The tea leaf roller and other three moths in the Family Gracillariidae are clustered into a branch of the phylogenetic tree with 100% bootstrap value (Figure 1). It infers that *C. theivora* is closely related to species of Gracillariidae, and the complete mitochondrial genome of *C. theivora* can be used for further taxonomic analysis.

**Figure 1.**
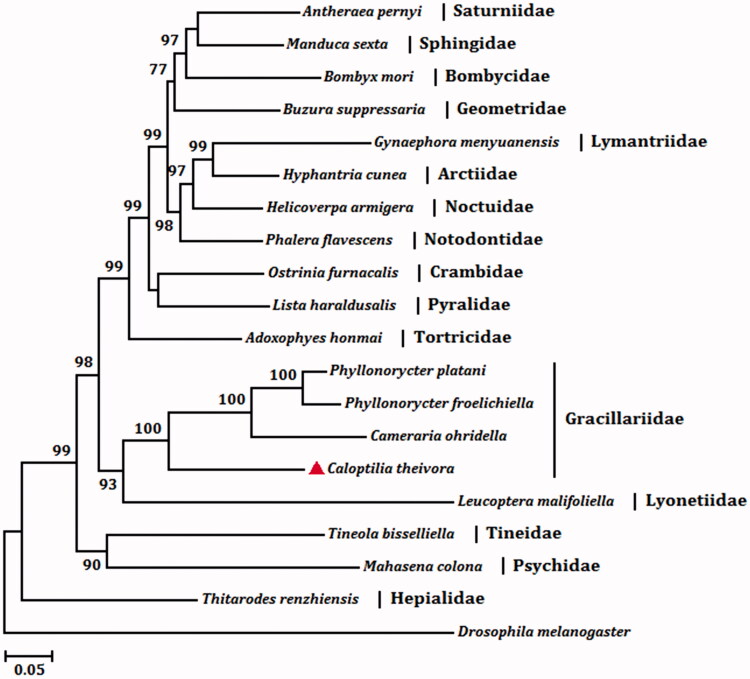
The maximum likelihood (ML) phylogenetic tree of *Caloptilia theivora* and other moths. The GenBank accession numbers used for tree constructed are as follows: *Mahasena colona* (KY856825), *Tineola bisselliella* (KJ508045), *Cameraria ohridella* (KJ508042), *Phyllonorycter platani* (KJ508044), *Phyllonorycter froelichiella* (KJ508048), *Buzura suppressaria* (KP278206), *Bombyx mori* (AF149768), *Antheraea pernyi* (HQ264055), *Manduca sexta* (EU286785), *Thitarodes renzhiensis* (HM744694), *Helicoverpa armigera* (GU188273), *Phalera flavescens* (JF440342), *Hyphantria cunea* (GU592049), *Gynaephora menyuanensis* (KC185412), *Leucoptera malifoliella* (JN790955), *Adoxophyes honmai* (DQ073916), *Ostrinia furnacalis* (AF467260), and *Lista haraldusalis* (KF709449).
